# Cardioprotection of tilianin ameliorates myocardial ischemia-reperfusion injury: Role of the apoptotic signaling pathway

**DOI:** 10.1371/journal.pone.0193845

**Published:** 2018-03-14

**Authors:** Cheng Zeng, Wen Jiang, Ruifang Zheng, Chenghui He, Jianguang Li, Jianguo Xing

**Affiliations:** 1 College of Pharmacy, Xinjiang Medical University, Urumqi, Xinjiang, P.R. China; 2 Xinjiang Institute of Materia Medica, Urumqi, Xinjiang P.R. China; 3 Department of Pharmacy, The Sixth Affiliated Hospital, Xinjiang Medical University, Urumqi, Xinjiang, P.R. China; University of Cincinnati College of Medicine, UNITED STATES

## Abstract

Our previous research demonstrated that tilianin protects the myocardium in a myocardial ischemia reperfusion injury (MIRI) rat model and has prominent pharmacological potential as a cardiovascular drug. Our study aimed to investigate the molecular signaling implicated in the improvement of myocardial survival induced by tilianin, a flavonoid antioxidant. Tilianin (2.5, 5, and 10 mg/kg/d) or saline was orally administered to rats for 14 days. On the 15th day, ischemia was induced by ligating the left anterior descending artery for 45 min, followed by 4 h of reperfusion. The levels of MIRI-induced serum myocardial enzymes and cardiomyocyte apoptosis as well as infarct size were examined to assess the cardioprotective effects. Cardiac tissues were collected for western blot analyses to determine the protein expression of anti-apoptotic signaling molecules. In MIRI-treated rats, our results revealed that pre-administration of high dose-tilianin the reduced release of LDH, MDA, and CK-MB and increased the plasma SOD level, and significantly attenuated the infarct size. Western blot analysis showed that a remarkable rise in expression of Bcl-2 and XIAP, and decline in expression of Bax, Smac/Diablo, HtrA2/Omi, cleaved caspase-3, caspase-7 and caspase-9 was observed in the myocardium. The apoptosis index of cardiomyocytes further supports the cardioprotective effect of tilianin. Additionally, compared with the MIRI model group, pretreatment with high dose-tilianin group upregulated phosphorylated Akt and PI3K. In contrast, using the PI3K inhibitor LY294002 to block Akt activation effectively inhibited the protective effects of tilianin against MIRI. Tilianin pretreatment was beneficial for activating the PI3K/Akt signaling pathway and inhibiting myocardial apoptosis.

## Introduction

Myocardial ischemia reperfusion injury (MIRI) is defined as tissue damage that occurs when early and fast coronary flow returns to the heart after ischemia, which often augments the myocardial injury [1–3]. During myocardial ischemia, there is a period where there is a lack of oxygen and nutrients, which will induce apoptotic cell death, and these conditions will further deteriorate during reperfusion. The principal cellular pathway leading to cardiomyocyte death is apoptosis [4]. In MIRI, excessive apoptosis causes the loss of cardiomyocyte volume and subsequent cardiac dysfunction [5]. Preventing apoptosis minimizes heart damage induced by MIRI [6]. One of the primary roles of apoptosis is the activation of a class of aspartate-specific cysteine proteases called caspases [7, 8]. Cells process multiple caspases, which may work in a cascade-like fashion. X-linked inhibitor of apoptosis protein (XIAP) potently prevents caspase activation [9, 10]. In addition, XIAP plays a significant role in cardiomyocyte apoptosis induced by MIRI [11, 12].

**Fig 1:**
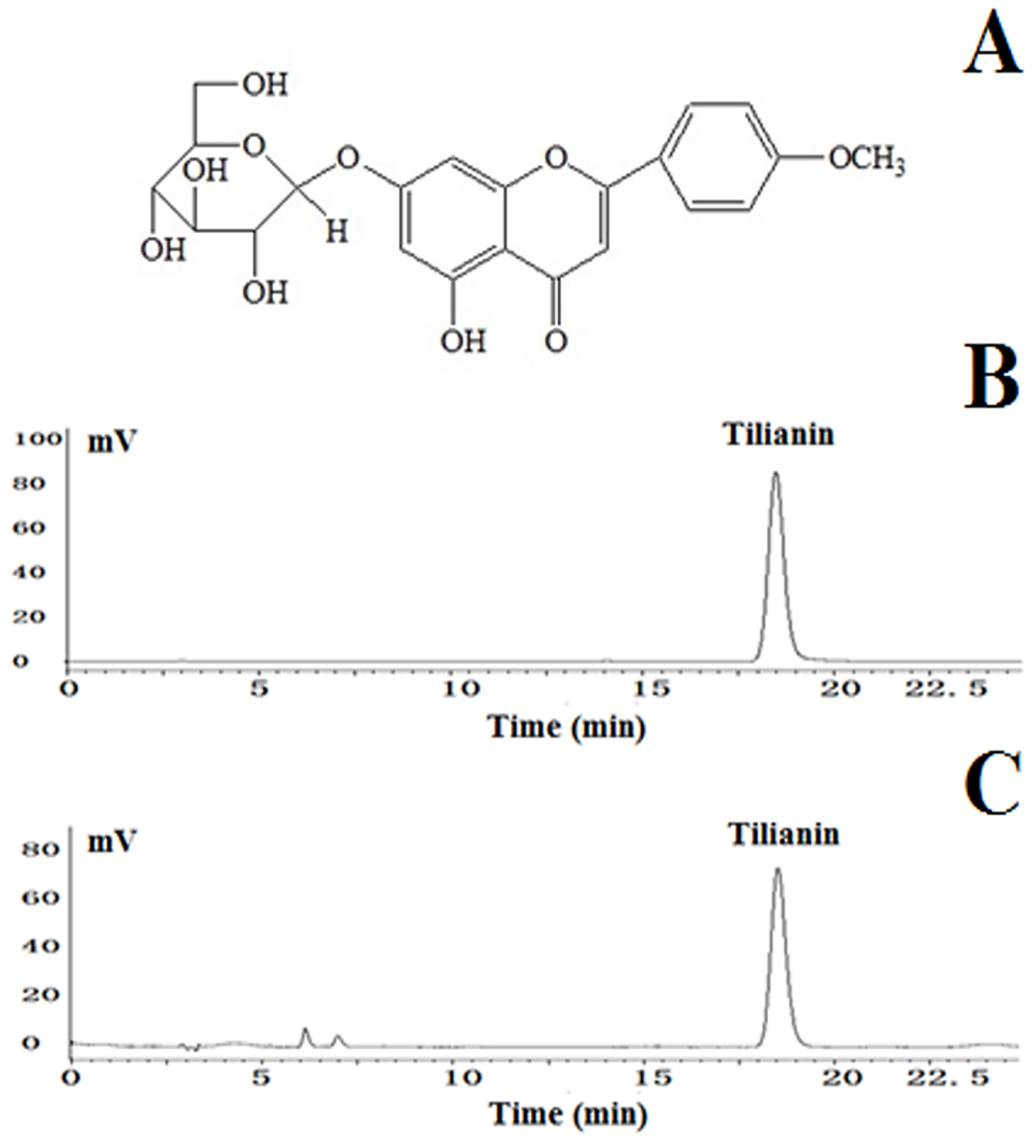
(A): Chemical structure of tilianin. Molecular weight: 446.4. Molecular formula: C_22_H_22_O_10_. (B): HPLC chromatogram of tilianin control. (C): HPLC chromatogram of tilianin sample.

Reducing the myocardial damage that occurs during the reperfusion period will efficiently reduce MIRI. However, while there are several effective methods that can be used to prevent reperfusion injury, further research is needed to acquire new methods and objectives to reduce MIRI. Currently, emphasis is placed on a variety of natural products that prevent and protect the myocardium against MIRI by activating the phosphatidylinositol 3-kinase (PI3K)/Akt signaling pathway. The used of these natural products has become a novel approach to the treatment of ischemic heart disease. The annual herb called Moldavian balm or Moldavian dragonhead, *Dracocephalum moldavica* L. (DML), has been used as an herbal medicine for more than 800 years in China [13–15]. In Uyghur folk medicine, granules of dried DML, called Badiranji Buya Keli, have been used clinically for a few hundred years to treat coronary heart disease, blood pressure, angina, neuralgia, and atherosclerosis [16, 17]. Commonly known as XinJiang in China, DML contains a potent antioxidant flavonoid component, tilianin (acacetin 7-glucoside, molecular formula: C_22_H_22_O_10_, molecular weight: 446.4, Fig 1A), which is used to prevent and treat hypertension and heart failure [12, 18]. Specifically, tilianin isolated from this medicinal herb has been shown to prevent MIRI in rats [19, 20] by conferring cardioprotection [21].

Until now, the cardioprotective effect of tilianin on MIRI and the underlying mechanism involved in the process has not been clear. Therefore, in the present study, we investigated whether pretreatment with tilianin reduced MIRI-induced apoptosis, elucidated the mechanisms of tilianin on myocardial apoptotic pathways in MIRI, and provided clinical references for the development of novel potential cardio-protective drugs (for the treatment of MIRI).

## Materials and methods

### Materials

Raw DML plants were obtained from the Xinjiang Institute of Materia Medica, Xinjiang, China. These plants were authenticated by Prof. Jiang He of Xinjiang University of Chinese Medicine. Radio-immunoprecipitation assay (RIPA) lysis buffer was obtained from Beyotime Institute of Biotechnology (Beijing, China). 2,3,5-Triphenyl tetrazolium chloride (TTC) was purchased from Sigma (Beijing, China). Malondialdehyde (MDA), lactate dehydrogenase (LDH), superoxide dismutase (SOD), and serum creatinine kinase MB (CK-MB) assay kits were purchased from Jiancheng Bioengineering Institute (Nanjing, China). LY294002 (PI3K inhibitor) was purchased from Abcam (Beijing, China). Terminal deoxynucleotidyl transferase dUTP nick-end labeling (TUNEL) assay kit was purchased from Boster Biological Technology co. Itd (Wuhan, China). The primary antibodies against phosphorylation of PI3K (p-PI3K), PI3K, phosphorylation of Akt (p-Akt), Akt, Bcl-2, Bax, caspase-3, cleaved caspase-3, caspase-7, Caspase-9, Smac/Diablo, HrtA2/Omi, XIAP, GAPDH, and β-actin were purchased from Cell Signaling Technology, Inc (CST, USA). Goat anti-rabbit secondary antibodies were purchased from the Zhongshan Company (Beijing, China).

### Preparation and analysis of tilianin

The herbal dry powders (500 g) were extracted three times in 40% aqueous ethanol (5000 mL) at room temperature. The extracted solution was filtered, and the alcohol in the combined filtrates was removed with rotary evaporator. The residue was partitioned with column chromatography using HPD600 resin and was eluted with water, 50% ethanol, and then 70% ethanol. The 70% ethanol eluent was filtered on a silica gel column (200–300 mesh, chloroform:methanol, 100:0→80:20) to remove impurities. The purified product was collected, and its structure was established by a combination of 1H-NMR and HPLC [21]. Its purity was determined by HPLC (Fig 1B and 1C) and was found to be greater than 97%; after drying, the product was reserved for further experimentation.

### Animal preparation and experimental design

Healthy male Sprague-Dawley (SD) rats (7–9 weeks) weighing between 280 and 320 g were purchased from Vital River Laboratories Co., Ltd. (Beijing, China). All animals were raised at room temperature (20–25°C) with 54±7% humidity and 12 h/12 h light/dark cycle (light on from 7:30 am to 7:30 pm). All rats had free access to food and water for a week before the experiment.

Ethical standards statement: This project was approved by the Department of Experimental Animal Center (DEAC) in Xuanwu Hospital of Capital Medical University (XHCMU), Beijing China. All animal experiments were approved by the Laboratory Animal Ethics Committee of the DEAC at the XHCMU (20170605–1205). Also, the operational procedures used in the animal experiments abide by national and institutional principles and protocols for the care and use of experimental animals.

### Experiment 1

The rats were randomly divided into five groups:

Group 1 (Sham group, n = 10): administrated normal saline (10 mL/kg, orally) for 14 days. On day 15, MIRI surgery was performed and thread was passed beneath the left anterior descending (LAD) coronary artery but the coronary artery was not occluded;
Group 2 (MIRI, n = 20): administrated normal saline (10 mL/kg, orally) for 14 days. On day 15, surgery was performed for LAD coronary artery ligation for 45 min following reperfusion for 4 h;
Group 3 (L-tilianin+MIRI, n = 20): administrated tilianin (2.5 mg/kg) for 14 days. On day 15, surgery was performed for LAD coronary artery ligation for 45 min following reperfusion for 4 h;
Group 4 (M-tilianin+MIRI, n = 20): administrated tilianin (5 mg/kg) for 14 days. On day 15, surgery was performed for LAD coronary artery ligation for 45 min following reperfusion for 4 h;
Group 5 (H-tilianin+MIRI, n = 20): administrated tilianin (10 mg/kg) for 14 days. On day 15, surgery was performed for LAD coronary artery ligation for 45 min following reperfusion for 4 h.

The five groups of rats were fed by gavage with the drug or normal saline for 14 days before surgery, at a volume of 10 mL/kg once a day (surgery was performed 15 min after the last administration).

### Experiment 2

Experiment 2 was conducted to test whether tilianin-induced XIAP expression in the myocardium was inhibited by LY294002 (a PI3K inhibitor). SD male rats were randomly separated into two groups:

Group 1 (H-tilianin+MIRI, n = 10): administrated tilianin (10 mg/kg) for 14 days. On day 15, surgery was performed for LAD coronary artery ligation for 45 min following reperfusion for 4 h;
Group 2 (H-tilianin+MIRI+ LY294002, n = 10): administrated tilianin (10 mg/kg) for 14 days. On day 15, surgery was performed for LAD coronary artery ligation for 45 min following reperfusion for 4 h;

The two groups of rats were fed by gavage with the drug or normal saline for 14 days before surgery, at a volume of 10 mL/kg once a day (surgery was performed 15 min after the last administration. In addition, rats in group 2 were pretreated with LY294002 (0.3 mg/kg in 5% DMSO) 30 min before reperfusion).

### Surgical procedures

All rats were subjected to tracheotomy and endotracheal intubation after intraperitoneal injection of pentobarbital sodium (40 mg/kg). Ventilation was performed by inserting a polyethylene-50 (PE-50) tube through the trachea and connecting it to a rodent ventilator with a tidal volume of 1.2 L/kg and breath rate of 70 /min. Body temperature was maintained at 37 °C using a heating pad. The heart rate and electrical activity of the heart were continuously monitored with an electrocardiogram (lead II configuration).

The chest was opened by a middle thoracotomy, followed by a pericardiotomy. To ligate the left anterior descending artery, the heart was exposed via a left lateral thoracotomy at the 4th intercostal space. A 6–0 Prolene loop along with a snare occlude were passed around the left coronary artery. Successful coronary occlusion was indicated when a visible blanched area that was distal to the ligation site was observed. The occlusion was maintained for 45 min followed by 4 h of reperfusion under ECG control. Successful reperfusion was demonstrated by a marked resolution of ST-elevation.

### Hemodynamic measurement

The ECG and heart rate of the experimental animals were recorded with a MP150 data acquisition system (BIOPAC Systems, Inc., USA). These parameters were recorded at the following time points corresponding to the basis of state (Baseline), ischemia for 45 min (Ischemia), and reperfusion for 4 h (Reperfusion).

### Myocardial infarction assessment

After 4 h of reperfusion, a total of 2 mL of 2% Evans Blue dye was injected into the aorta. All of the rats were euthanized by cervical dislocation. The hearts were quickly removed, washed, frozen, and cut into approximately five cross sections (each 2–3 mm thick) from the apex of the junction site. Myocardial infarct areas in the left ventricle were detected via the TTC method. Heart tissues were incubated in 1% TTC in phosphate-buffered saline (PBS, pH 7.4) at 37°C for 15 min. After 3 washes in PBS, tissues were fixed for 12 h in 10% formalin solution to increase the contrast. The area stained blue by Evans Blue is the area not at risk. The area stained red by TTC represents ischemic but viable tissue. Infarcted myocardium was not stained by either TTC or Evans Blue and is paler than TTC stained area. The area of infarct size (IS) and area at risk (AAR) were quantitated using Image-Pro Plus 6.0 software (Media Cybernetics, Inc., USA). IS and AAR were expressed as percentages of the left ventricular (LV) area (IS/AAR and AAR/LV, respectively).

### Cardiac function measurement

Echocardiography was conducted after 4 h of reperfusion. Briefly, the rats were anesthetized and sedated (2% isoflurane), and two-dimensional echocardiography was studied using an echocardiography (GE ViVid 7.0, General Electric Company, USA) with a 20-MHz probe. All measurements represent the mean of 5 consecutive cardiac cycles. The left ventricular ejection fraction (LVEF) and left ventricular fractional shortening (LVFS) were automatically calculated by computer algorithms. All of these measurements were performed in a blinded manner.

### Myocardial apoptosis determination

Apoptosis of cardiomyocytes was determined by TUNEL staining. First, the myocardia were fixed in 4% paraformaldehyde, embedded in paraffin, and then sectioned (2 μm). TUNEL staining was performed, and the individual nuclei were visualized with an optical microscope at a magnification of 400×. ImagePro Plus 6.0 software was used to calculate the percentage of TUNEL-positive nuclei (positive nuclei/total nuclei), with at least 10 randomly chosen fields from each slide for statistical analysis.

The experiments were carried out in a blinded manner. The data were calculated by formula [22]:

$$\text{The}\;\text{apoptotic}\;\text{index}=\frac{\text{number}\;\text{of}\;\text{TUNEL}\;\text{positivenuclei}}{\text{number}\;\text{of}\;\text{total}\;\text{nuclei}}\times \text{100\%}$$

### Biochemical studies

Cardiomyocyte injury was assessed by measuring serum LDH and CK-MB levels. At the end of reperfusion, the serum LDH and SOD activities, and MDA content were measured by spectrophotometry. CK-MB was quantitated using a commercially available ELISA kit according to the manufacturer’s instructions.

After the blood was collected, the heart samples were removed, rapidly placed in cold saline (4 °C), and then stored at -80°C. The heart homogenate (weight of heart: volume of buffer = 1:7) was prepared with ice-cold radio-immunoprecipitation assay (RIPA) buffer and then centrifuged at 5500 × *g* and 4 °C for 15 min. The supernatant was used for determination of total protein with BCA Protein Assay kit (Beyotime Institute of Biotechnology, Haimen, China).

### Western blot analysis

The tissue homogenate (equal to 50 μg protein tissue) and cell supernatant were boiled in loading buffer (containing 1% phenylmethanesulfonyl fluoride (PMSF)) for 10 min and then subjected to sodium dodecyl sulfate-polyacrylamide gel electrophoresis (SDS-PAGE) on 10% and 12% gels. Then, targeted proteins were electrotransferred onto PVDF membranes (Millipore, Bedford, MA, USA). The membranes were washed with TBST five times, 10 min each time. The membranes were incubated in 5% dry milk for 2 h and then overnight (24 h) at 4°C with the following primary antibodies: p-PI3K (1:500), PI3K (1:1000), p-Akt (1:500), Akt (1:1000), XIAP (1:200), cleaved caspase-3 (1:1000), caspase-7 (1:1000), caspase-9 (1:1000), Bcl-2 (1:1000), Bax (1:1000), HtrA2/Omi (1:1000), Smac/Diablo (1:1000), β-actin (1:1000) and GAPDH (1:1000). After an additional five washes, the corresponding horse radish peroxidase-conjugated goat anti-rabbit secondary antibodies (1:2000) were added and incubated with the membranes for 3 h at room temperature. Protein bands were visualized using an enhanced chemiluminescence (ECL; Thermo Fisher Scientific Inc., USA) kit and quantitated by densitometric analysis using AlphaView SA software (3.4.0.0, ProteinSimple, USA). GAPDH and β-actin were used as internal reference proteins.

### Statistical analysis

The data are presented as the mean ± standard error (SD). Statistical analysis was performed by evaluating one-way analysis of variance (ANOVA), followed by Turkey’s test method using SPSS version 20.0 software. Values were considered statistically significant when *P* <0.05.

## Results

### Exclusion and mortality

A total of 110 rats were initially enrolled into the study. Two rats died from anesthetic accidents, and 4 died during early reperfusion. A further three rats with ligation and relaxation of the coronary arteries were excluded due to infarct size <10% or technical failure of TTC staining. Complete data sets were obtained from the remaining 101 rats.

### Effect of tilianin on the electrocardiogram (ST segment) of MIRI rats

**Fig 2:**
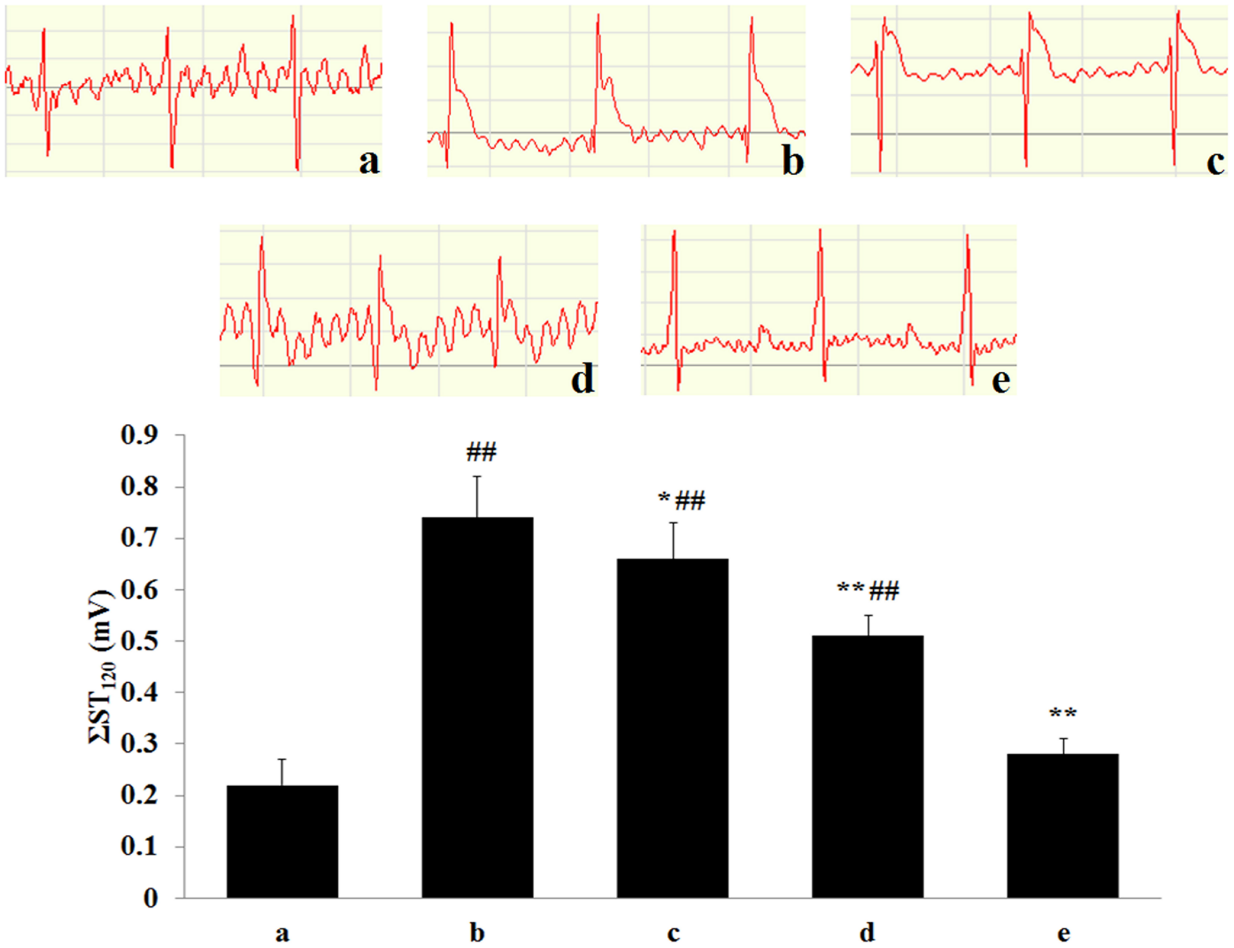
Effect of pre-administration with tilianin on ST-segment elevation after MIRI in rats. Rats were subjected to 45 min of ischemia followed 4 h of reperfusion was measured. (Effect of tilianin on ST-segment elevation. Results are expressed as mean ± SD. The traces of ECG in various groups, a: Sham; b: MIRI; c: L-tilianin (2.5 mg/kg); d: M-tilianin (5 mg/kg) and e: H-tilianin (10 mg/kg). ^##^*P*<0.01 vs. the Sham group;*P<0.05, **P<0.01 vs. the MIRI group, n = 10).

As shown in Fig 2, compared with the Sham group, the ST segments of the MIRI model group were significantly increased (*P*<0.01), which indicated that the MIRI model was successfully implemented. However, compared with the MIRI group, pretreatment with H-tilianin significantly inhibited the elevation of ΣST (*P*<0.01).

### Effects of tilianin on myocardial enzymes and oxidative stress markers

In Table 1, compared with the Sham group, MIRI group has a noticeable elevation in LDH and CK-MB activation (*P*<0.01). Compared with the MIRI group, all serum myocardial enzymatic activities were significantly reduced in the tilianin-pretreated groups (LDH: MIRI+M-tilianin and MIRI+H-tilianin, *P*<0.01; CK-MB: MIRI+M-tilianin and MIRI+H-tilianin, *P*<0.01).

**Table 1:** Influence of tilianin on LDH, CK-MB, SOD and MDA in rat hearts after MIRI (n = 10).

Groups	LDH (U/mL)	CK-MB (ng/mL)	SOD (U/mL)	MDA (nmol/mL)
**Sham**	45.39±4.81	2.48±0.32	23.27±4.05	3.31±0.62
**MIRI**	279.66±41.26^ΔΔ^	6.39±0.89^ΔΔ^	11.38±1.99^ΔΔ^	8.54±1.82^ΔΔ^
**L-tilianin**	251.29±19.48*	5.98±0.61*	13.33±1.54*	7.32±0.75*
**M-tilianin**	161.15±8.71**	4.76±0.28**	17.58±1.84**	4.29±0.35**
**H-tilianin**	58.38±5.29**	2.54±0.23**	24.63±3.73**	2.51±0.47**

^ΔΔ^*P* < 0.01 vs Sham;

*P < 0.05,

**P < 0.01 vs MIRI

Compared with the Sham group, the SOD activity was markedly reduced and MDA content was significantly increased following MIRI in the MIRI group (*P*<0.01). Compared with the MIRI group, preconditioning with different concentrations of tilianin significantly decreased the MDA content (MDA: MIRI+M-tilianin and MIRI+H-tilianin, *P*<0.01), by contrast, tilianin pretreatment with different concentrations increased the SOD activity at the end of the reperfusion (SOD: MIRI+M-tilianin and MIRI+H-tilianin, *P*<0.01).

### Myocardial infarct size

**Fig 3:**
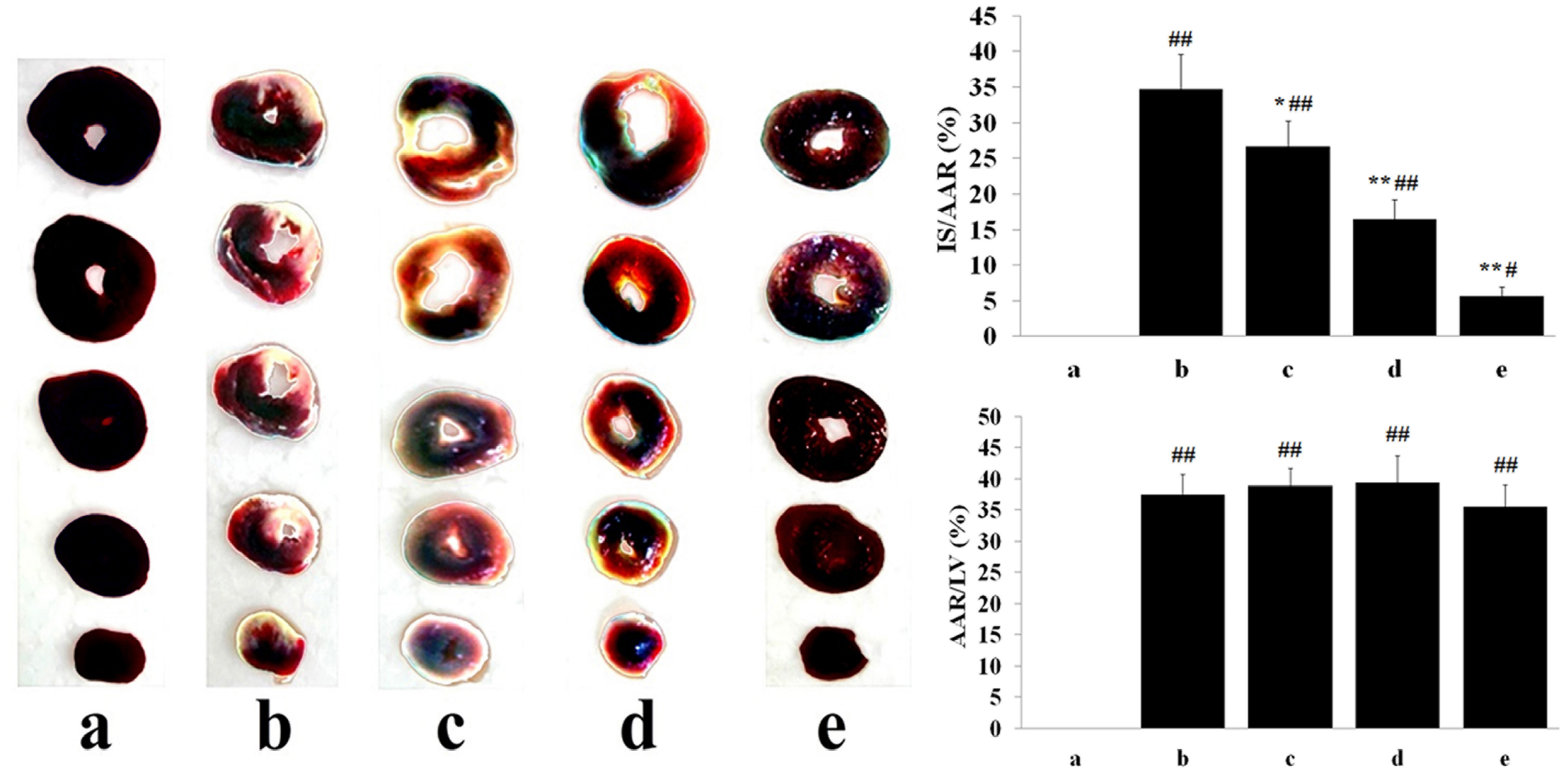
Images of TTC and Evans Blue stained heart sections, and the quantitative analysis. (a: Sham; b: MIRI; c: L-tilianin; d: M-tilianin; e: H-tilianin, ^#^*P*<0.05, ^##^*P*<0.01 vs. the Sham group;*P<0.05, **P<0.01 vs. the MIRI group, n = 10).

As shown in Fig 3, the cardiac tissue slices of the Sham group showed no obvious infarction. Compared with the Sham group, the MIRI group exhibited significant myocardial infarct areas (34.71±4.82, vs. 0%, *P*<0.01), which were reduced by pretreating with different concentrations of tilianin. Compared with the MIRI group rats, myocardial infarct sizes were significantly reduced in M-tilianin-(16.42±2.31%, *P*<0.01) and H-tilianin-(5.62±1.35%, *P*<0.01) rats. In addition, Compared with MIRI group, the AAR/LV in other groups did not change significantly (*P*>0.05).

### Determination of cardiac function

**Fig 4:**
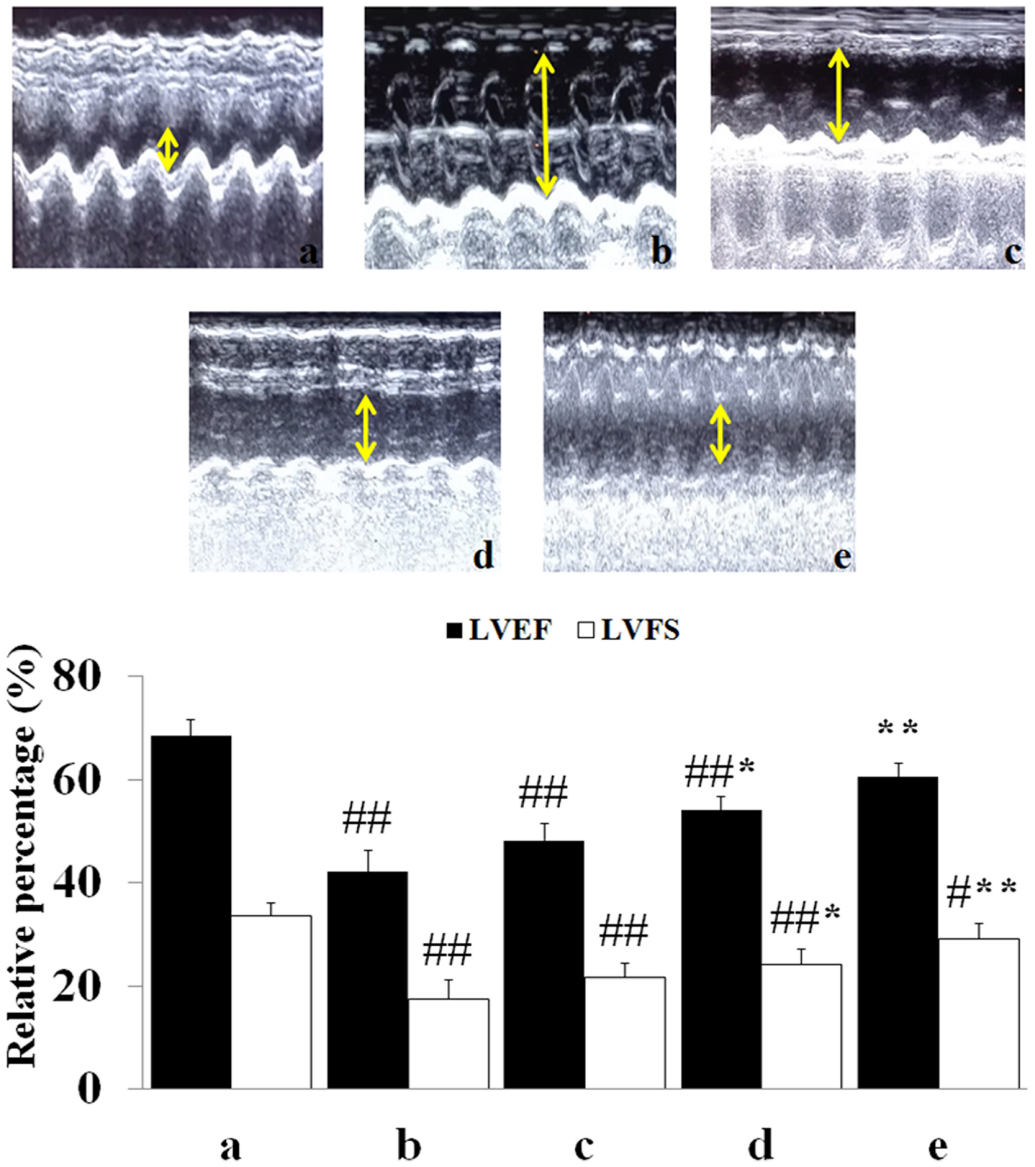
Echocardiographic analysis. (a: Sham; b: MIRI; c: L-tilianin; d: M-tilianin; e: H-tilianin, ^#^*P*<0.05, ^##^*P*<0.01 vs. the Sham group;*P<0.05, **P<0.01 vs. the MIRI group, n = 10).

As seen in Fig 4, compared with the sham group, the LVEF and LVFS significantly decreased in MIRI group (P<0.01). Meanwhile, compared with the MIRI group, M- and H-tilianin group had a significant increasing effect on the LVEF and LVFS (*P*<0.01).

### Effect of tilianin on myocardium apoptosis

**Fig 5:**
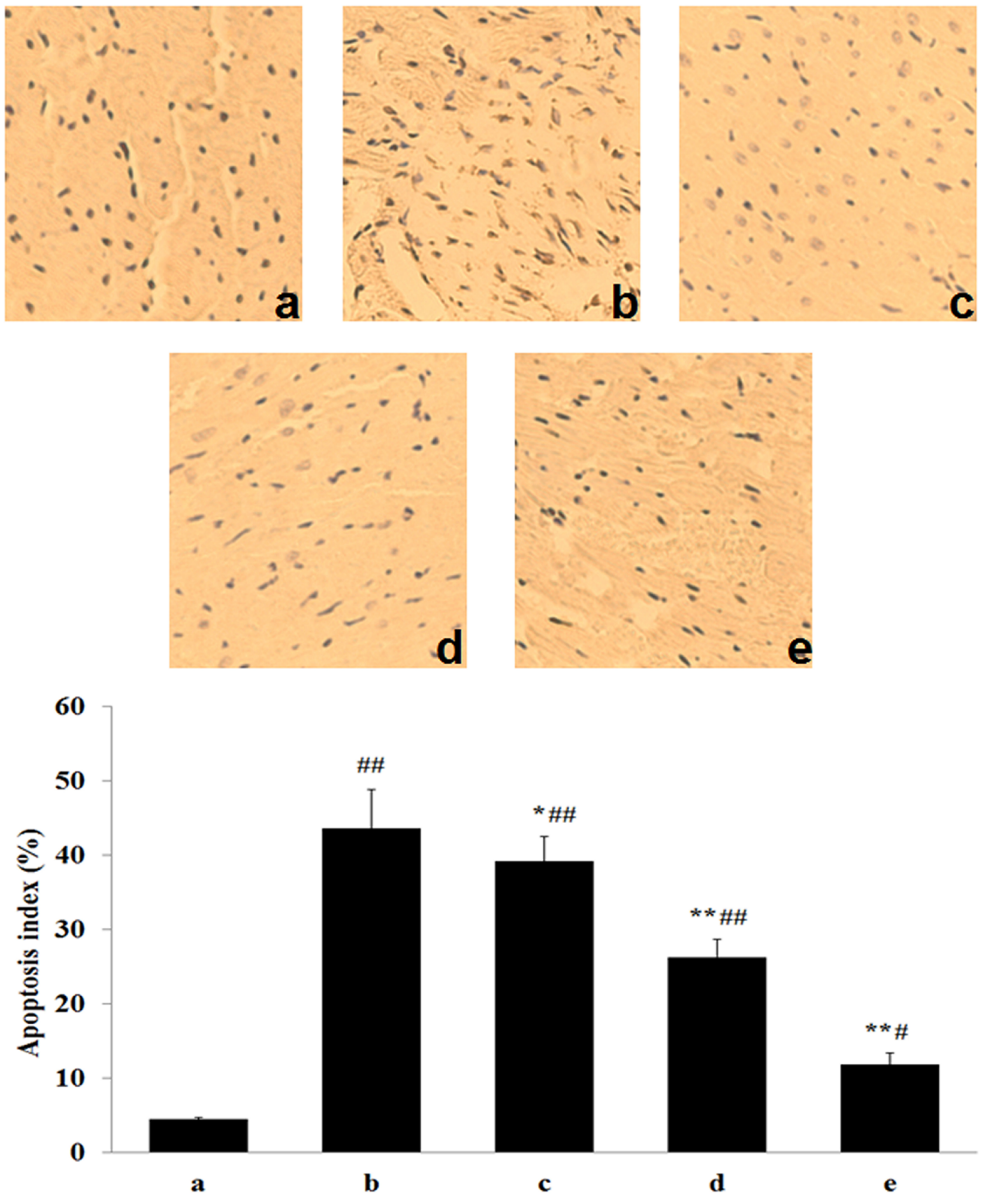
Detection apoptotic myocytes by TUNEL assay in each group. Representative images from Sham group, MIRI group and various administrations are shown at 400× magnifications. (a: Sham; b: MIRI; c: L-tilianin; d: M-tilianin and e: H-tilianin, ^#^*P*<0.05, ^##^*P*<0.01 vs. the Sham group;*P<0.05, **P<0.01 vs. the MIRI group, n = 10).

In Fig 5, apoptotic cardiomyocyte were identified by TUNEL analysis, apoptotic cardiomyocyte appears brown stained whereas TUNEL-negative appears blue. TUNEL staining to detect apoptosis showed that MIRI induced an observable increase in the percentage of TUNEL-positive cells caused by apoptosis (Sham: 1.7%±0.3%; MIRI: 43.8%±6.8%; *P*<0.01). However, in the MIRI+H-tilianin group, the increase in the percentage of TUNEL-positive cells was observably less than that in the MIRI group (12.7%±2.5% vs 43.8%±6.8%; *P*<0.01).

### Western blotting

**Fig 6:**
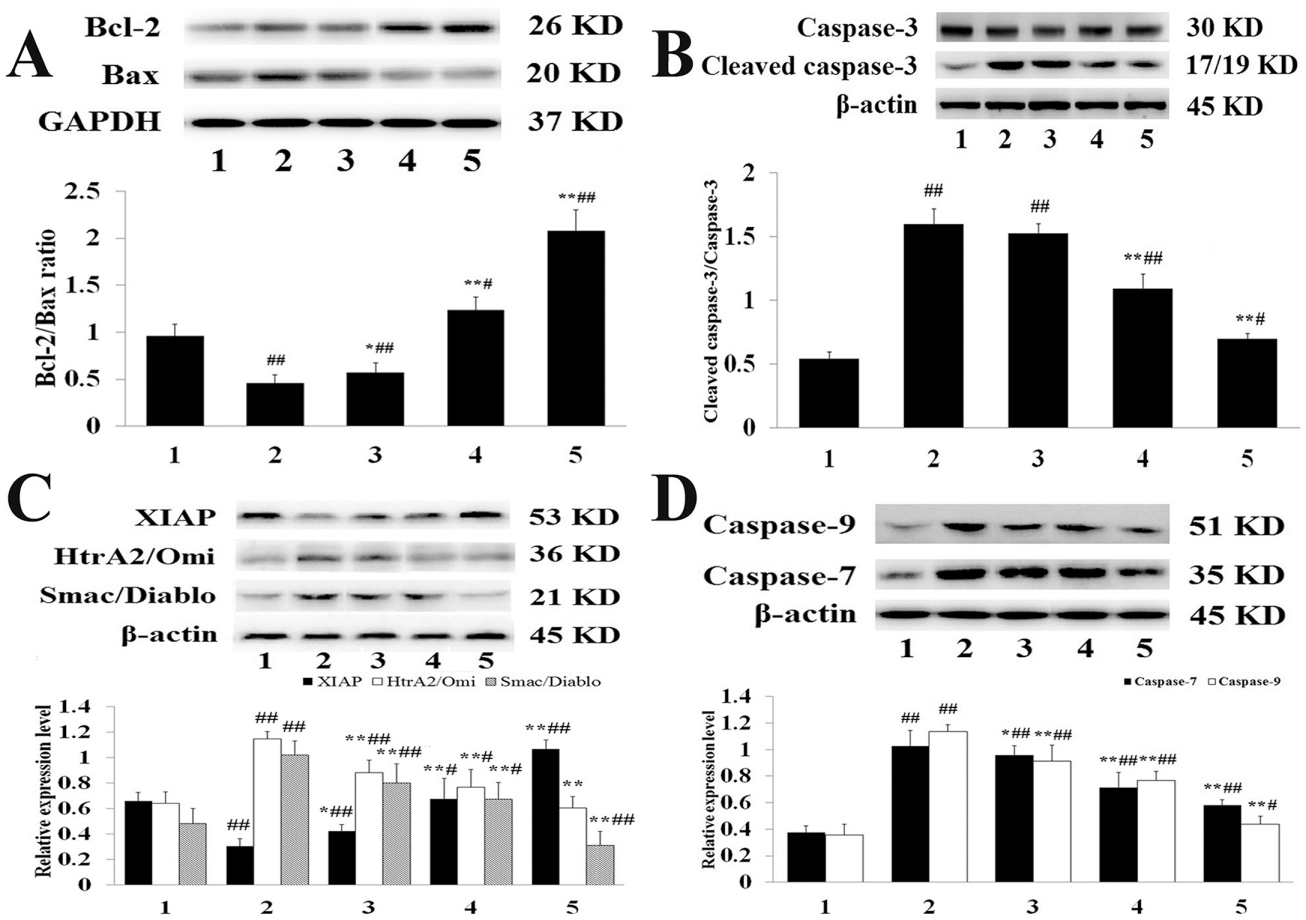
**(A): Bcl-2, Bax and GAPDH expression levels in the myocardial tissue of each group**. (1: Sham, 2: MIRI, 3: L-tilianin, 4: M-tilianin and 5: H-tilianin) (^#^*P*<0.05, ^##^*P*<0.01 vs. the Sham group;*P<0.05, **P<0.01 vs. the MIRI group, n = 10). **(B): Cleaved-caspase-3, caspase-3 and β-actin expression levels in the myocardial tissue of each group**. (1: Sham, 2: MIRI, 3: L-tilianin, 4: M-tilianin and 5: H-tilianin) (^#^*P*<0.05, ^##^*P*<0.01 vs. the Sham group; **P<0.01 vs. the MIRI group, n = 10). **(C): XIAP, HtrA2/Omi, Smac/Diablo and β-actin expression levels in the myocardial tissue of each group**. (1: Sham, 2: MIRI, 3: L-tilianin, 4: M-tilianin and 5: H-tilianin) (^#^*P*<0.05, ^##^*P*<0.01 vs. the Sham group;*P<0.05, **P<0.01 vs. the MIRI group, n = 10). **(D): Caspase-7, Caspase-9 and β-actin expression levels in the myocardial tissue of each group**. (1: Sham, 2: MIRI, 3: L-tilianin, 4: M-tilianin and 5: H-tilianin) (^#^*P*<0.05, ^##^*P*<0.01 vs. the Sham group;*P<0.05, **P<0.01 vs. the MIRI group, n = 10).

The expression levels of Bcl-2, Bax, and cleaved caspase-3 are shown in Fig 6A and 6B. Compared with the Sham group, MIRI clearly induced the expression of Bax and cleaved caspase-3 (*P*<0.01). Meanwhile, compared with the MIRI group, these were elevated expression of Bcl-2 but decreased Bax and cleaved caspase-3 expression in the H-tilianin group (*P*<0.01), and the effect of tilianin was dose-dependent. In addition, as illustrated in Fig 6C, compared with the MIRI group, pre-administration with M- and H-tilianin significantly reduced the expression of HtrA2/Omi and Smac/Diablo and increased the expression of XIAP (*P*<0.01).

To confirm the effect of tilianin, the expression levels of specific cell apoptosis markers, including caspase-7 and caspase-9, were analyzed (Fig 6D). Compared with the Sham group, the expression of caspase-7 and caspase-9 significantly increased in the MIRI group (*P*<0.01). It was demonstrated that the activities of all the indicated markers were significantly enhanced in the MIRI group, representing the activation of apoptosis during MIRI induction. Meanwhile, compared with the MIRI group, the expression of caspase-7 and caspase-9 significantly reduced in the H-tilianin group (*P*<0.01). The reduction of apoptosis by tilianin was verified by the inhibition of activation of all the apoptosis-specific indicators in myocardial tissues in the group that was pre-treated with tilianin.

**Fig 7:**
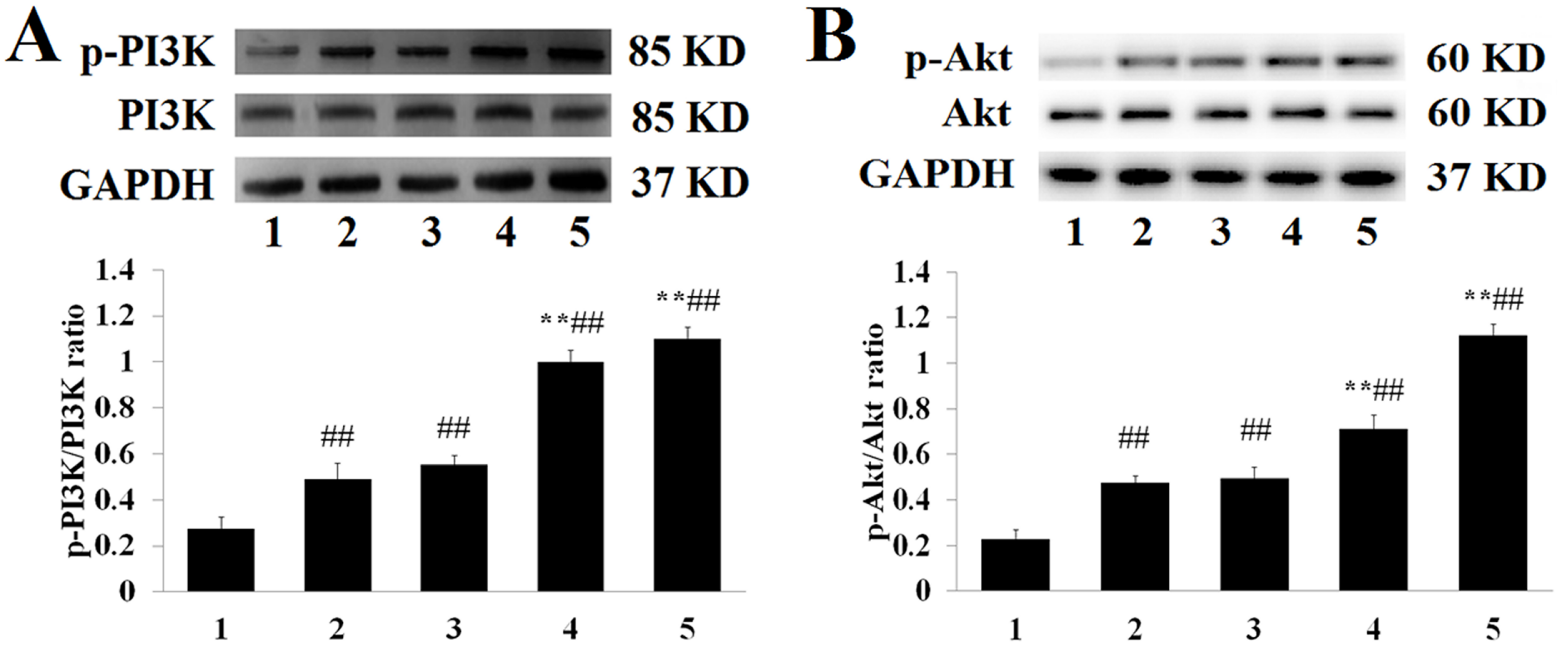
**(A): p-Akt, Akt and GAPDH expression levels in in the myocardial tissue of each group**. (1: Sham, 2: MIRI, 3: L-tilianin, 4: M-tilianin, 5: H-tilianin) (^##^*P*<0.01, **P<0.01, n = 10). **(B): p-PI3K, PI3K and GAPDH expression levels in in the myocardial tissue of each group**. (1: Sham, 2: MIRI, 3: L-tilianin, 4: M-tilianin, 5: H-tilianin) (^##^*P*<0.01 vs. the Sham group; **P<0.01 vs. the MIRI group, n = 10).

To further explore the underlying mechanism that results in tilianin attenuating apoptosis caused by MIRI treatment, the expression of proteins within the PI3K/Akt pathway was investigated in groups. As shown in Fig 7A and 7B, MIRI group underwent a dramatic increased in the expression of p-Akt (phosphorylation of Akt), p-PI3K (phosphorylation of PI3K), p-PI3K/PI3K and p-Akt/Akt ratios, whereas there was no apparent effect on the level of total Akt and PI3K (*P*>0.05). Compared with the MIRI group, in pre-administrated with tilianin groups, the p-Akt, p-PI3K, p-Akt/Akt and p-PI3K/PI3K ratios gradually returned to a relatively high level, and the effect was dose-dependent. In addition, the activity of Akt can also be represented by levels of HtrA2/Omi, Smac/Diablo, and XIAP.

### LY294002 inhibits H-tilianin-induced activation of the PI3K/Akt pathway

**Fig 8:**
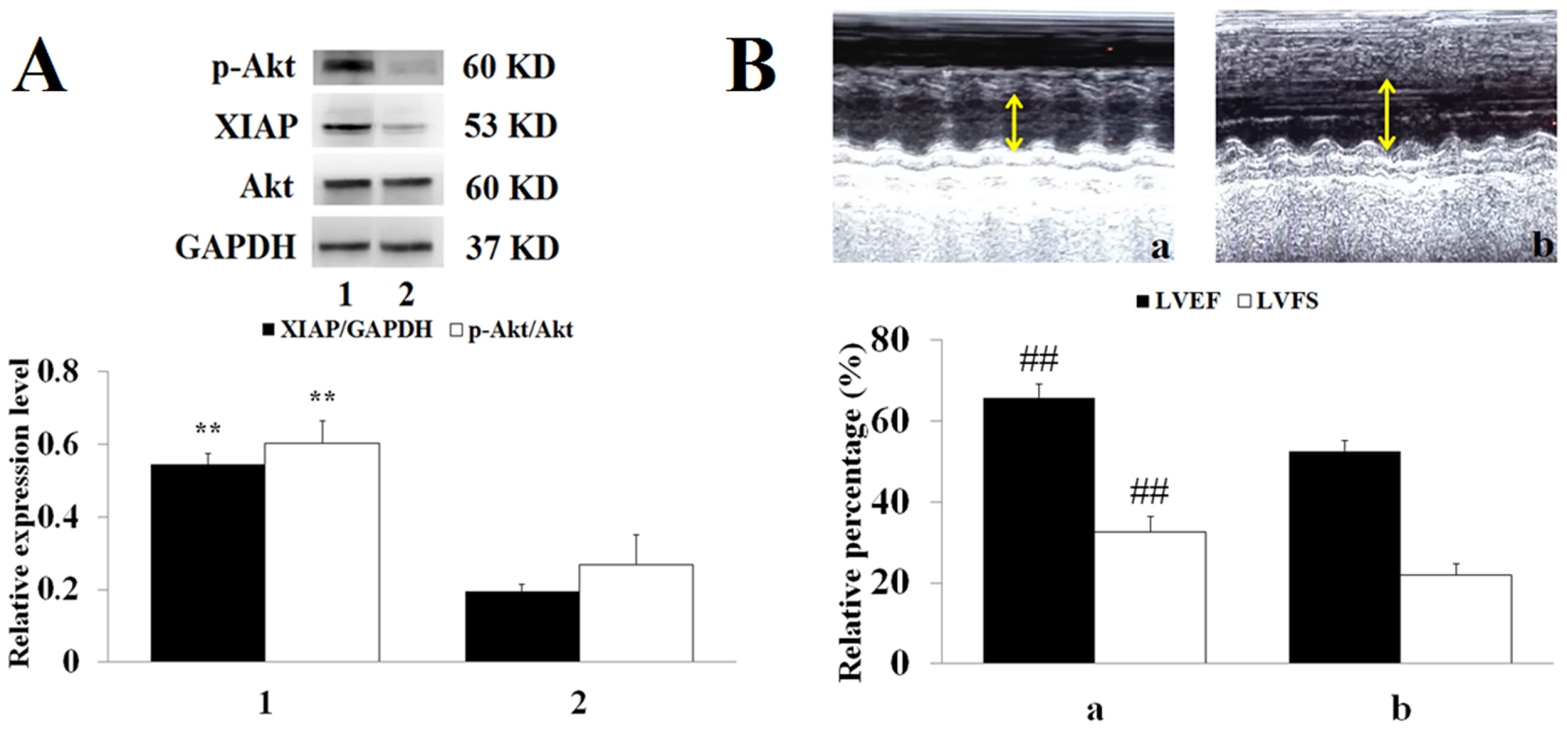
**(A): p-Akt, XIAP, Akt and GAPDH expression levels in the myocardial tissue of each group**. (a: MIRI+H-tilianin and b: MIRI+H-tilianin+LY294002) (**P<0.01 vs. the MIRI+H-tilianin+LY294002 group, n = 10). **(B): Echocardiographic analysis (a: MIRI+H-tilianin and b: MIRI+H-tilianin+LY294002)**. (**P<0.01 vs. the MIRI+H-tilianin+LY294002 group, n = 10).

In experiment 2 (Fig 8A and 8B), the role of the PI3K/Akt signaling pathway in the protection of MIRI via tilianin pre-treatment was clarified. Compared with the MIRI+H-tilianin group, p-Akt and XIAP protein levels were observably reduced and the LVEF and LVFS were significantly decreased in the MIRI+H-tilianin+LY294002 group (*P*<0.01). Additionally, the up-regulated effect of tilianin was blocked by the combined use of LY294002. As shown in Table 2, compared with the H-tilianin+MIRI group, co-treatment with LY294002 increased LDH activity (*P*<0.01), CK-MB activity (*P*<0.01) and MDA content (*P*<0.05), and attenuated SOD activity (*P*<0.05).

**Table 2:** Effect of MIRI+H-tilianin and MIRI+H-tilianin+LY294002 groups on the levels of LDH, CK-MB, SOD and MDA (n = 10).

Groups	LDH (U/mL)	CK-MB (ng/mL)	SOD (U/mL)	MDA (nmol/mL)
**1**	56.74±3.44^ΔΔ^	2.65±0.26^ΔΔ^	21.75±3.53^Δ^	2.72±0.71^Δ^
**2**	232.8±25.62	5.85±0.75	16.24±1.65	6.83±2.29

1: MIRI+H-tilianin; 2: MIRI+H-tilianin+LY294002;

^Δ^*P* < 0.05,

^ΔΔ^*P* < 0.01 vs MIRI+H-tilianin+LY294002

## Discussion and conclusion

Our previous pharmacokinetic research showed that the plasma concentration of tilianin solution increased with time over 15 min and decreased slowly to 12 h [19]. In this study, we adopted *in vivo* animal models to investigate the protective effect of tilianin against MIRI. We found that tilianin pretreatment conferred a cardioprotective effect, as evidenced by improvement after cardiac injury and reduced myocardial apoptosis. Importantly, PI3K/Akt signaling was proven to play a key role in this process. This study indicates that tilianin protects against myocardial tissue damage in MIRI rats.

During MIRI, the integrity of the membrane of the myocardial muscle cells is damaged, causing increased permeability of the cell membrane and the release of myocardial enzymes into the blood, which significantly increases the content of serum enzymes including LDH, CK-MB, MDA, and SOD. LDH and CK-MB are key myocardial enzymes used to estimate the degree of myocardial injury. Pre-treatment with high-dose tilianin (10 mg/kg) significantly inhibited CK-MB and LDH levels, indicating that tilianin may protect against MIRI. Additionally, we found that tilianin led to a reduction of MDA and enhanced SOD activity, affirming the anti-oxidative properties of tilianin [23, 24].

Apoptosis is mediated by two different evolutionarily conserved pathways: the intrinsic and extrinsic cell death pathways, which are respectively represented by the Bcl-2 family and the caspase family [25, 26]. Thus, in the apoptotic pathway, the Bcl-2 family proteins, consisting of death antagonists (Bcl-2) and agonists (Bax), are pivotal regulatory components. These critical players are part of the three major pathways of apoptosis. The extrinsic apoptotic pathway mainly involves death receptor Bcl-2/Bax mediation and activation of caspase-8 [27, 28]. The intrinsic pathway is mediated via mitochondrial dysfunction and activation of caspase-9 [29]. The last apoptosis pathway is mediated through the activation of caspase-12 [30]. Ischemia, especially when combined with reperfusion, triggers translocation of Bax into the outer mitochondrial membrane, which is related to elevated Bax levels and a reduced Bcl-2/Bax ratio [31]. It has been shown that overexpression of Bcl-2 observably decreases cardiomyocyte apoptosis and reduces the myocardial infarct area after MIRI [32]. Our results suggest that tilianin pre-treatment significantly elevated Bcl-2 and decreased Bax, which corresponded to the increased Bcl-2/Bax ratio. Based on these findings, we speculated that changes in the ratio of pro-apoptotic to anti-apoptotic proteins may also contribute to tilianin’s observed anti-apoptotic mode of action [33].

Among numerous proteins that are known to regulate caspase activity, XIAP is the most potent endogenous inhibitor of apoptosis [34]. Particularly, XIAP binds to caspase-3, caspase-7, and caspase-9, a process that may be regulated by Smac/Diablo (a mitochondrial protein and negative regulator of XIAP) and HtrA2/Omi (an inhibitor of XIAP) [35]. In our study, we found that oral tilianin pre-treatment before ischemia/reperfusion reduced XIAP degradation. This was most likely achieved by restraining the Smac/Diablo and HtrA2/Omi protease activity, resulting in ameliorated myocardial function, reduced myocardial infarct area, and restrained proteolytic processing of caspase-7 and caspase-9. Thus, our data provides strong evidence that reduced mitochondrial leakage of Smac/Diablo and HtrA2/Omi and increased XIAP expression summarily led to decreased caspase activity during MIRI.

Earlier research has shown that the PI3K/Akt signaling pathway is a classical pro-survival and anti-apoptosis pathway, which is central to mitigating MIRI [36]. Akt activation has been shown to have a beneficial effect on the MIRI heart [37]. In our study, we used LY294002 prior to reperfusion to assess whether activation of the PI3K/Akt pathway would mechanistically promote tilianin-induced cardioprotection. Tilianin-induced p-Akt and XIAP expression in the myocardium was inhibited by LY294002, suggesting that tilianin upregulates myocardial XIAP expression by the PI3K/Akt signaling pathway.

**Fig 9:**
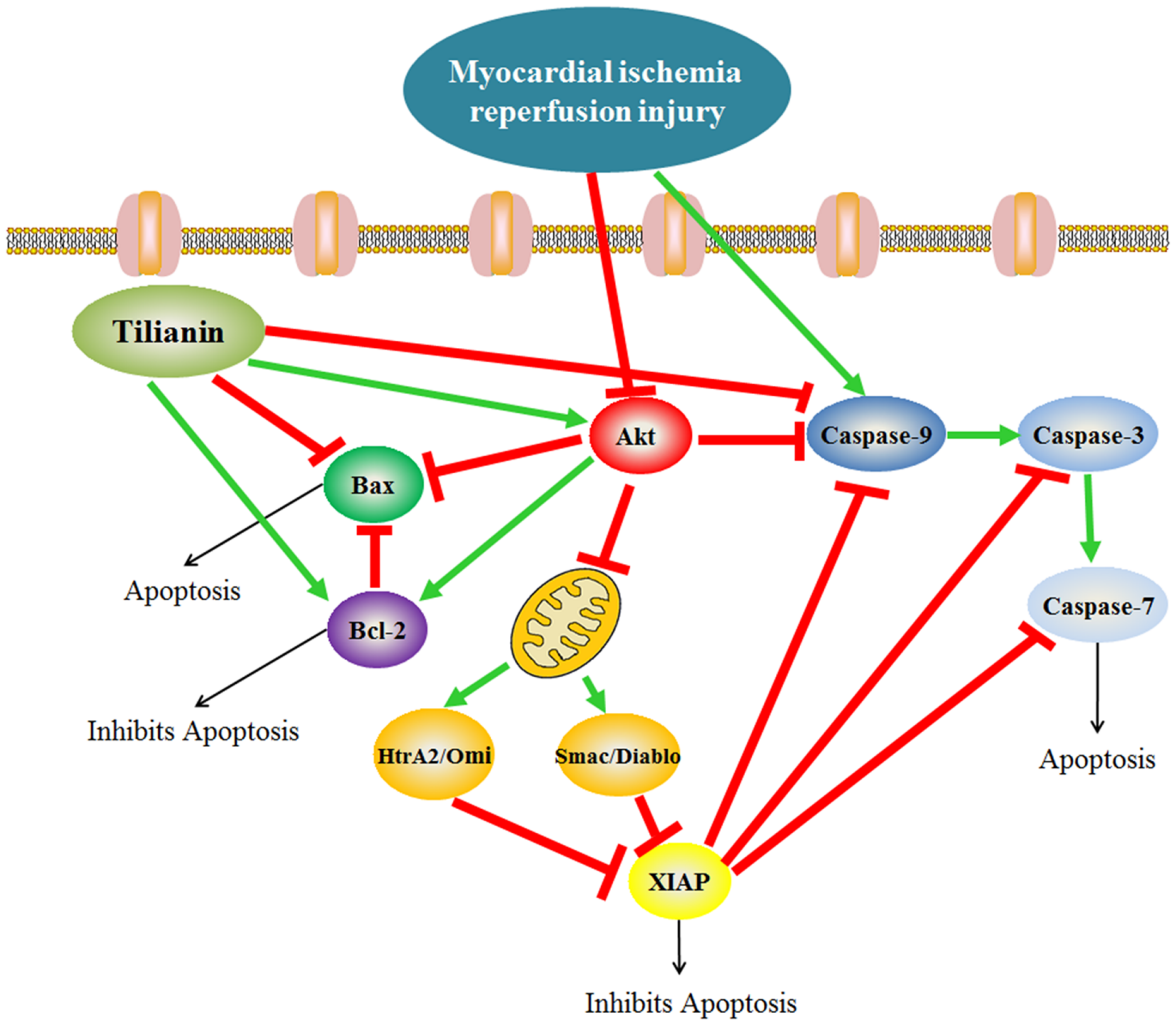
Putative mechanism of tilianin improved myocardial apoptosis. (Tilianin pretreatment significantly activated PI3K/Akt pathway and inhibited release of Smac/Diablo and HrtA2/Omi from mitochondria, XIAP degradation and caspase activation).

Herein, the *in vivo* cardioprotective function of tilianin against MIRI was investigated. Our findings showed that oral administration of tilianin not only decreased infarct size and scavenging free radicals but also inhibited myocardial apoptosis. Thus, tilianin may exude cardio-protection via an anti-apoptotic signaling pathway (Fig 9). Additionally, tilianin might function through activation of the PI3K/Akt pathway and then inhibition of MIRI-induced apoptosis through inhibiting Smac/Diablo and HtrA2/Omi, and activating XIAP.
